# Shrinkage and Mechanical Properties of Self-Compacting SFRC With Calcium-Sulfoaluminate Expansive Agent

**DOI:** 10.3390/ma13030588

**Published:** 2020-01-27

**Authors:** Changyong Li, Pengran Shang, Fenglan Li, Meng Feng, Shunbo Zhao

**Affiliations:** 1 International Joint Research Lab for Eco-building Materials and Engineering of Henan, North China University of Water Resources and Electric Power, Huauyuan Campus, No. 36 Beihuan Road, Zhengzhou 450045, China; lichang@ncwu.edu.cn (C.L.); lifl64@ncwu.edu.cn (F.L.); 2 School of Civil Engineering and Communication, North China University of Water Resources and Electric Power, Huanyuan Campus, No. 36 Beihuan Road, Zhengzhou 450045, China; z201810312234@stu.ncwn.edu.cn; 3 Henan Provincial Collaborative Innovation Center for Water Resources High-efficient Utilization and Support Engineering, North China University of Water Resources and Electric Power, Longzihu Campus, No. 136 Jinshui East Road, Zhengzhou 450046, China

**Keywords:** self-compacting SFRC, volume fraction of steel fiber, expansive agent, mix proportion design, workability, shrinkage, mechanical properties

## Abstract

With the premise of ensuring workability on a fresh mixture, the volume stability of hardened self-compacting steel fiber reinforced concrete (SFRC) becomes an issue due to the content of cementitious materials increased with the volume fraction of steel fiber. By using the expansive agent to reduce the shrinkage deformation of self-compacting SFRC, the strength reduction of hardened self-compacting SFRC is another issue. To solve these issues, this paper performed an experimental investigation on the workability, shrinkage, and mechanical properties of self-compacting SFRC compared to the self-compacting concrete (SCC) with or without an expansive agent. The calcium-sulfoaluminate expansive agent with content optimized to be 10% mass of binders and the steel fiber with a varying volume fraction from 0.4% to 1.2% were selected as the main parameters. The mix proportion of self-compacting SFRC with expansive agent was designed by the direct absolute volume method, of which the steel fibers are considered to be the distributed coarse aggregates. Results showed that rational high filling and passing ability of fresh self-compacting SFRC was ensured by increasing the binder to coarse-aggregate ratio and the sand ratio in the mix proportions; the autogenous and drying shrinkages of hardened self-compacting SFRC reduced by 22.2% to 3.2% and by 18.5% to 7.3% compared to those of the SCC without expansive agent at a curing age of 180 d, although the expansion effect of expansive agent decreased with the increasing volume fraction of steel fiber; the mechanical properties, including the compressive strength, the splitting tensile strength, and the modulus of elasticity increased with the incorporation of an expansive agent and steel fibers, which met the design requirements.

## 1. Introduction

To overcome the casting difficulty of concrete in structural members and joints with complex shapes and dense reinforcements, and to reduce the environmental pollution of vibration noise, a high-performance self-compacting steel fiber reinforced concrete (SFRC) becomes more and more applicable in engineering structures [1,2,3,4]. Self-compacting SFRC not only realizes the self-compacting performance with high passing and filling workability without segregation and bleeding, but also overcomes the brittleness of self-compacting concrete (SCC) by using steel fibers [5,6,7].

Based on literatures reported, many previous works sacrificed the workability of SCC to inhibit the shrinkage by using the constant mix proportion of base materials without adjusting with the increase of volume fraction of steel fiber. Aslani and Nejadi [8] reported that the drying shrinkage of SCC was reduced by 9.3% at 364 d by directly admixing hooked-end steel fiber (circular section, *l*_f_ = 60 mm, *d*_f_ = 0.75 mm) with volume fraction *v*_f_ = 0.38%, but the diameter of J-ring flow was decreased by 11.5%. Corinaldesi and Moriconi [9] reported that by admixing the hooked-end steel fiber (circular section, *l*_f_ = 30 mm, *d*_f_ = 0.7 mm) with volume fraction *v*_f_ = 0.64%, the drying shrinkage of SCC decreased about 37.5%, while the time *T*_50_ of slump-flow to diameter of 50 cm and the elapsed time to gain the final configuration increased respectively by 50% and 30%. Grabois et al. [10] studied the self-compacting lightweight concrete which prepared with coarse and fine lightweight aggregates by adding hooked-end steel fiber (circular section, *l*_f_ = 35 mm, *d*_f_ = 0.55 mm) with volume fraction *v*_f_ = 0.5%, and the drying shrinkage decreased about 7%, but the V-funnel flow time increased by 30 s. Meng and Khayat [11] investigated the effect of hybrid fibers on properties of ultra high-performance concrete, of which the straight steel fiber (circular section, *l*_f_ = 13 mm, *d*_f_ = 0.2 mm) and the hooked-end steel fiber (circular section, *l*_f_ = 30 mm, *d*_f_ = 0.5 mm) were used. Results showed that the autogenous shrinkage reduced by 30% with the increase of *v*_f_ from 2% to 5%, but the V-funnel time increased by 61.5%. Bensaci et al. [12] found that although the drying shrinkage of self-compacting SFRC reduced by 33% with the *v*_f_ increased from 0% to 1%, the workability became bad with an increase of the *T*_50_ by 81.4%. Therefore, these studies did not take into account the shrinkage performance of hardened self-compacting SFRC with the premise of ensuring workability on a fresh mixture. This produces the hidden issue in casting and molding quality of self-compacting SFRC. One of the risks is the strength reduction of self-compacting SFRC. In the study of Bensaci et al. [12], the cubic compressive strength decreased by 9.36% with the *v*_f_ having increased from 0% to 1.5%. Grabois et al. [10] also found that the V-funnel flow time of self-compacting lightweight concrete increased 30 s by using steel fiber with a volume fraction of *v*_f_ = 0.5%, and the cylindrical compressive strength decreased by 14.3%. Khaloo et al. [13] reported that with the increasing *v*_f_ from 0% to 2% of hooked-end steel fiber (rectangular section, *l*_f_ = 20.4 mm), the slump-flow reduced from 800 mm to 640 mm, while the cylindrical compressive strength reduced by 18.6%.

In order to improve the workability of self-compacting SFRC, the mix proportion should be adjusted. El-Dieb and Reda [14] studied the effect of fiber factor (the product of the aspect ratio with the volume fraction) and cementitious material content on properties of fresh mixture. Results indicated that with the maximum fiber factor of 50, 90, and 100, the cement content of mixtures should increase to 350 kg/m^3^, 400 kg/m^3^ and 500 kg/m^3^, respectively. By keeping a constant thickness of mortar wrapped on the fibers and coarse-aggregates, Khayat et al. [15,16] produced the self-compacting SFRC with high filling and passing ability and sufficient stability, of which the cementitious materials was 475 kg/m^3^, and the dosage of coarse-aggregate was reduced 8.8% with the *v*_f_ = 0.5%. Ding et al. [17,18] proposed a method to get a result of indirect reduction of the coarse-aggregate content by regarding steel fibers as coarse-aggregates, of which the binders’ content and sand ratio were increased with the volume fraction of steel fiber. However, with the premise of ensuring the workability of a fresh mixture by adjusting mix proportion, the hardened self-compacting SFRC faces a problem of great shrinkage compared with SCC due to the content of cementitious materials increased with the volume fraction of steel fiber [16,17,18,19,20]. An experimental study exhibited that to keep the diameter of slump-flow at 600 mm for the self-compacting SFRC with the *v*_f_ of hooked-end steel fiber (circular section, *l*_f_ = 30 mm, *d*_f_ = 0.5 mm) increased from 0% to 1.4%, the binders content was increased by 16.3% and the dosage of coarse-aggregate was reduced by 35%. In this condition, the autogenous and drying shrinkages respectively increased by 19.8% and 53% [21].

In general, the contradictory changes of workability and shrinkage of self-compacting SFRC are difficult to harmonize by only adjusting the contents of base materials of SCC with steel fibers. The greater shrinkage of self-compacting SFRC is needed to be controlled by a means of adding an expansive agent [22,23]. Su et al. [24] reported that the drying shrinkage of ultra high-performance concrete was reduced 29.5% by using a calcium–magnesium composite expansive agent. Choi et al. [25] found that the shrinkage of alkali activated material mortar was reduced by 23.1% due to admixing calcium-sulfoaluminate expansive agent. However, this also raises another problem of the reduction of compressive strength of SCC. He et al. [26] pointed out that when the dosage of calcium-aluminate expansive agent was less than 9%, the cubic compressive strength of SCC decreased by 9.6%. Li et al. [27] found that the cubic compressive strength of SCC decreased by 11.5% by adding a calcium-sulfoaluminate and calcium-oxide composite expansive agent. This mainly comes down to the transversal deformation with the unconfined expansion of SCC specimens. Therefore, the presence of steel fibers in SCC could confine the expansion [28,29], the expansion rate abated 81.5% of self-compacting SFRC with steel fiber (circular section, *l*_f_ = 35 mm, *d*_f_ = 0.55 mm) of *v*_f_ = 0.75% compared to that of SCC under the standard curing condition at (20 ± 2) °C temperature and *RH* ≥ 95%, while a slight decrease of the expansion rate at about 0.07% of self-compacting SFRC with the *v*_f_ = 0.25–0.75% was produced under the sealed cure and top-surface exposure curing conditions. uAfroughsabet et al. [30] presented that the conventional SFRC with K-type expansive cement was improved by 28.4% of cylindrical compressive strength and 39.2% of splitting tensile strength with the *v*_f_ = 1.0% hooked-end steel fibers, while the expansion was fully cancelled due to the inhibition effect of fibers. Geng et al. [31] also reported that to ensure the workability of a fresh mixture by increasing 7% binder and reducing 8.8% coarse-aggregate, the drying shrinkage of conventional SFRC with the *v*_f_ = 0.8% increased by 22.2%, which could not be removed by adding expansive agent. This means that a coordination exists between the dosage of expansive agent and the content of steel fiber to get the balance of expansion and shrinkage for self-compacting SFRC.

Based on the above analyses, the workability, shrinkage, and strength of self-compacting SFRC are complexly affected by the proportion of base materials of SCC, the content of steel fiber, and the addition of expansive agent. For engineering application, the study should be done on the premise of a rational workability of self-compacting SFRC with different volume fraction of steel fiber, and then the effect of considered parameters on basic mechanical properties and shrinkage of self-compacting SFRC should be verified to satisfy the design requirements. According to this technical route, this paper investigates the workability, mechanical properties, and shrinkage of self-compacting SFRC with calcium-sulfoaluminate expansive agent. The optimal dosage of the expansive agent was firstly determined, and the volume fraction of steel fiber was selected as the main parameter. The mix proportion of self-compacting SFRC was designed with the absolute volume method, of which steel fibers are considered as the distributed coarse aggregates. Results are discussed combined with the influencing mechanisms of studied properties.

## 2. Experimental Works

### 2.1. Raw Materials

Ordinary Portland cement in strength grade of 42.5, fly ash of class-II and limestone powder were used as binder. The chemical compositions are presented in Table 1, of which LOI is the loss on ignition. The physical and mechanical properties are presented in Table 2 and Table 3. Their properties met the relevant specifications of China codes GB 175, GB/T 1596 and GB/T 35164 [32,33,34].

**Figure 1:**
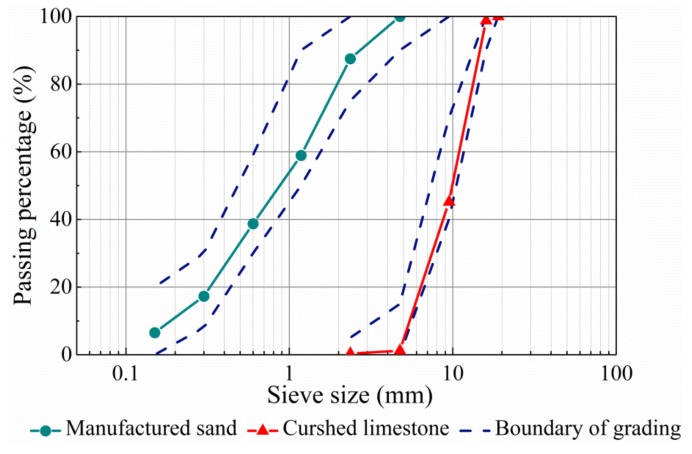
Grading of fine and coarse aggregates.

**Figure 2:**
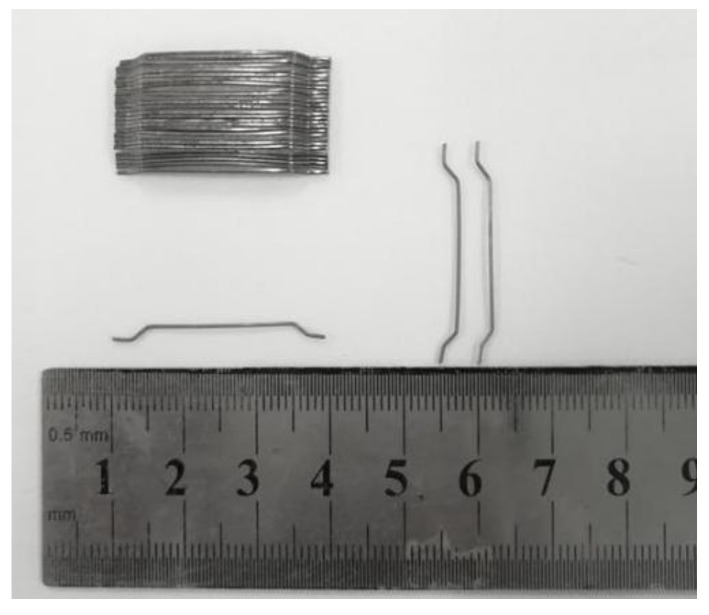
Type and size of steel fiber.

The fine aggregate was manufactured sand with a fineness modulus of 2.9 and the density of 2689 kg/m^3^. The coarse aggregate was crushed limestone with a maximum particle size of 16 mm and density of 2766 kg/m^3^. The water absorption of fine and coarse aggregates was 2.0% and 1.18% respectively, which were considered to adjust the water dosage of the mixture. The grading curves of fine and coarse aggregates presented in Figure 1 met the specifications of China codes GB/T 14684 and GB/T 14685 [35,36]. The steel fiber has hook-ends with the circular section as exhibited in Figure 2, the length *l*_f_ = 29.8 mm and the diameter *d*_f_ = 0.5 mm, the aspect ratio *l*_f_/*d*_f_ = 60, the tensile strength was 1150 MPa.

The calcium-sulfoaluminate expansive agent was used, the properties met the requirement of China code GB 23439 [37]. The optimal dosage of expansive agent was 10% mass of binder materials on the analysis of experimental results about the compressive strength and restrained expansion rate of cement mortar. Polycarboxylate superplasticizer with the water-reducing ratio up to 25% and the solid content of 21% was also used. Both of them were produced by Jiangsu Sobute New Materials Co. Ltd. of China (Nanjing, China). The mixing water was tap-water.

### 2.2. Mix Proportions

In this study, the main parameter was the volume fraction of steel fiber *v*_f_ = 0.4%, 0.8% and 1.2%. The strength grade of concrete was 40 MPa, and the target cubic compressive strength was 46.6 MPa. Based on previous studies [17,18,19], the water-to-binder ratio *w/b* = 0.31, the fly ash and limestone powder were added to the replacement of binder materials by mass of 10% and 20%, respectively. The mix proportions were determined by using the absolute volume method, results are shown in Table 4, of which the binder to coarse-aggregate ratio and sand ratio increased with the *v*_f_, while the dosage of coarse aggregate decreased. The SCC trial was used as a reference without expansive agent, the SF0 was the SCC with expansive agent, the SF4, SF8, and SF12 were self-compacting SFRC with expansive agent and *v*_f_ = 0.4%, 0.8% and 1.2% respectively.

### 2.3. Test Methods of Workability

The slump-flow and J-ring tests by the method of reversal slump cone for the workability of fresh SCC and self-compacting SFRC were conducted in accordance with EFNARC [38], ASTM C1611/C1611M-09 [39] and China codes JGJ/T 283 [40] and CECS 13 [41]. The indexes of the diameter of slump-flow (*D*), the time of slump-flow to diameter of 50cm (*T*_50_), and the difference of diameter between slump-flow and J-ring flow (*D*-*D*_J_) were selected to evaluate the filling and passing ability of fresh mixture. The initial target values are shown in Table 5.

### 2.4. Tests for Shrinkage Properties

**Figure 3:**
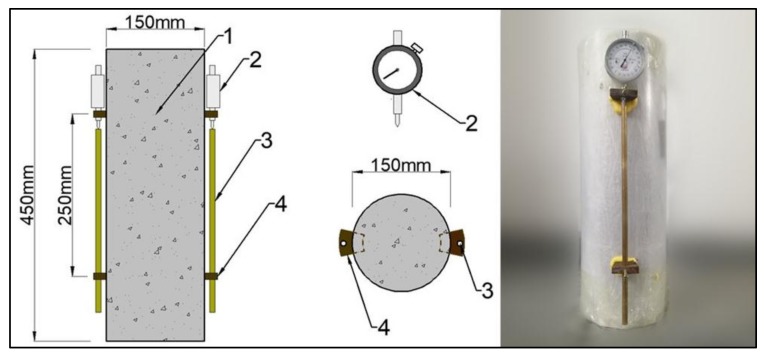
Sketch of specimens and measuring device for autogenous shrinkage deformation. 1—Cylinder specimen; 2—Dial indicator; 3—Gauge copper rod; 4—Embedded parts.

The deformation of autogenous shrinkage was measured by using the dial indicator with accuracy of 0.001 mm. The cylindrical specimens of Φ150 mm × 450 mm, two of them as a trial, were casted with special steel formwork. After casting and until demolding, the surfaces of specimens were covered by a waterproof plastic film to prevent the evaporation of water. All specimens were demolded after 24 h, and then sealed with polyester film and PVC sleeve immediately. As presented in Figure 3, the embedded parts were fixed in the reserved grooves on both sides of specimens with epoxy resin, the gauge copper rods and dial indicators were installed, and then the initial readings were measured. All specimens were tested in a chamber with constant temperature of (20 ± 2) °C and relative humidity *RH* = (60 ± 5)%. Autogenous shrinkage was calculated by Equation (1):

(1) $$\varepsilon_{\mathrm{as},t}\;=\;\left(\varepsilon_{t}-\varepsilon_{0}\right)/L_{b}$$

where, *ε*_as,t_ is the autogenous shrinkage (με) of specimen at *t* (d) age which computed initially at the specimen moved into the chamber; *ε*_t_ is the reading of dial indicator at *t* (d) age; *ε*_0_ is the initial reading of dial indicator; *L*_b_ is the distance between two embedded parts, *L*_b_ = 250 mm in this test.

**Figure 4:**
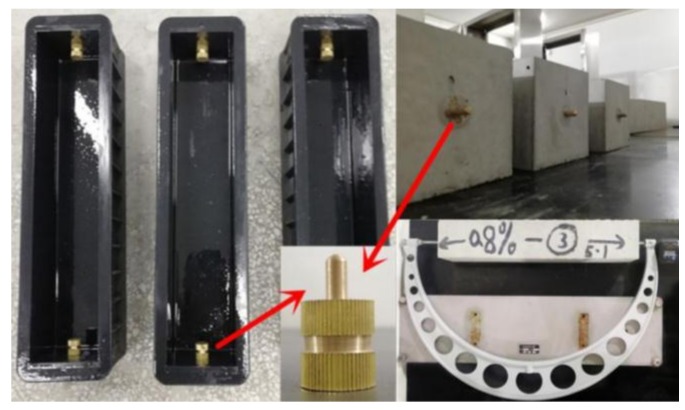
Measuring of drying shrinkage for self-compacting SFRC.

The drying shrinkage was measured in accordance with the China code JG/T 472 [42]. The prism specimens of 100 mm × 100 mm × 515 mm were used, three of them were tested as a trial. As displayed in Figure 4, the copper probes were embedded in ends of the mold before casting. After casting and until demolding, the surface of specimens was covered by a waterproof plastic film to prevent the evaporation of water. All specimens were demolded after 24 h and cured in a standard curing room with temperature of (20 ± 2) °C and *RH* of 95% for 2 days, and then moved into a chamber with constant temperature of (20 ± 2) °C and *RH* of (60 ± 5)%, while the initial length of specimen was measured immediately. The length of specimen was measured by using outside micrometer with accuracy of 0.001 mm. Drying shrinkage was calculated by Equation (2):

(2) $$\varepsilon_{\mathrm{ds},t}=(L_{t}-L_{0})/\left(L_{0}-2b\right)$$

where, *ε*_ds,t_ represents the drying shrinkage deformation (με) of specimen at *t* (d) age which computed initially at the specimen moved into the chamber; *L*_t_ is the length of specimen at *t* (d) age; *L*_0_ is the initial length of specimen; *L*_0_ = 515 mm; *b* is the embedded depth of copper probe, *b* = 22.5 mm in this test.

### 2.5. Tests for Mechanical Properties

Test methods of mechanical properties of self-compacting SFRC were in accordance with China code JG/T 472 [42], ASTM C39 [43] and BS EN 12390-3-2009 [44]. For each mixture of self-compacting SFRC, six cubic specimens with dimensions of 150 mm and six cylinders with dimensions of Φ150 mm × 300 mm were made. Three of the cubic specimens as a trial were tested respectively for the cubic compressive strength and the splitting tensile strength. Three of the cylinders as a trial were respectively tested for the axial compressive strength and the modulus of elasticity. All specimens were demolded after 24 h and cured in a standard curing room with temperature of (20 ± 2) °C and *RH* of 95% for 28d before testing.

## 3. Results and Discussion

### 3.1. Workability of Fresh Self-Compacting SFRC

**Figure 5:**
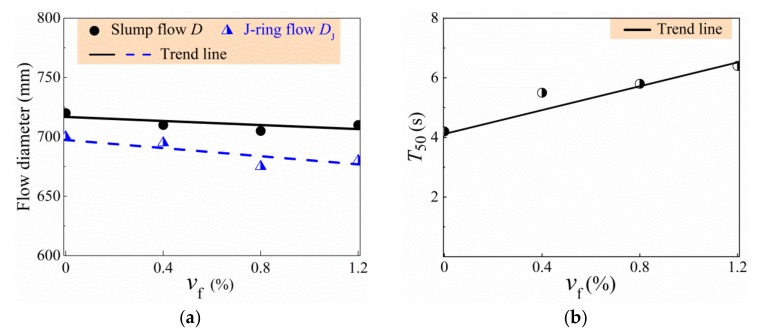
Variations of workability indexes with *v*_f_: (**a**) Diameter of slump-flow and J-ring flow; (**b**) *T*_50_.

Test results are exhibited in Table 5 and displayed in Figure 5. Generally, the workability of self-compacting SFRC characterized by the diameter of slump-flow (*D*), the time of slump-flow to a diameter of 50 cm (*T*_50_), and the difference of diameter between slump-flow and J-ring flow (*D*-*D*_J_) met the requirement of target values. Moreover, no segregation and bleeding were observed with the increase of sand ratio, and the high filling and passing ability of self-compacting SFRC was ensured by increasing the binder to coarse-aggregate ratio and the sand ratio. Comparing the values between SF0 and SCC, a slight influence of expansive agent can be seen to have existed on the workability of SCC. With the *v*_f_ increased from 0.4% to 1.2%, the *D*_J_ decreased by 2.9% and the *T*_50_ increased by 16.4%, while the *D-D*_J_ increased from 20 mm to 30 mm, indicating that the viscosity increased and the passing ability decreased by the presence of steel fibers; however, the slump-flow did not vary obviously due to the increase of binder to coarse-aggregate ratio. According to EFNARC [38], the slump-flow of self-compacting SFRC can be classified as SF2-class (660–750 mm). According to China code JGJ/T 283 [40], the workability of self-compacting SFRC met the PA1-level with *D*-*D*_J_ lower than 50 mm.

### 3.2. Autogenous Shrinkage of Hardened Self-Compacting SFRC

**Figure 6:**
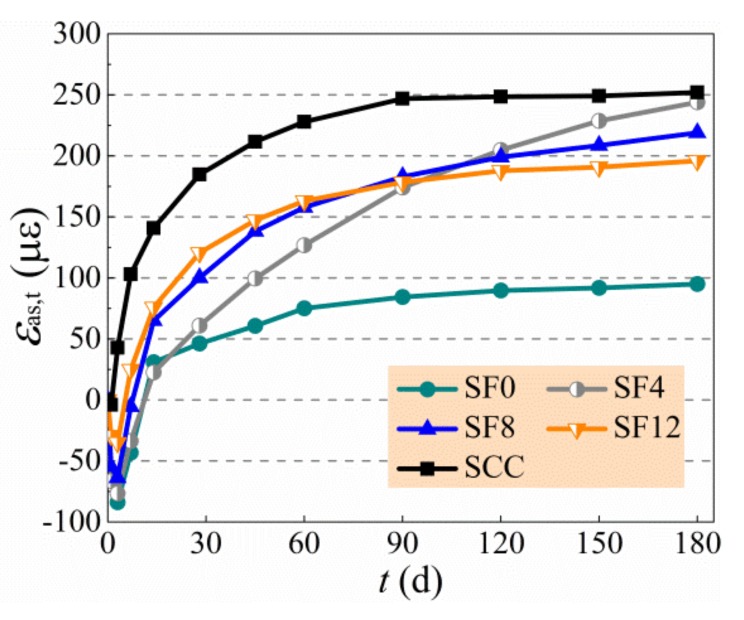
Autogenous shrinkage of tested specimens.

**Figure 7:**
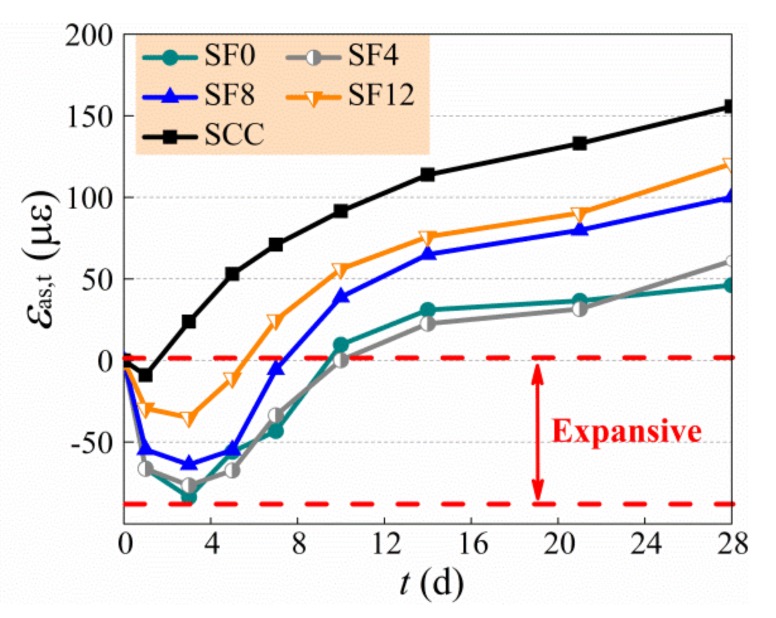
Changes of expansive deformation with the *v*_f_.

The test results of autogenous shrinkage of specimens are exhibited in Table 6 and Figure 6, and the growth of expansive deformation is displayed in Figure 7, of which the negative and positive values of *Y*-axis are the expansive and shrinkage deformation, respectively. In general, the autogenous shrinkage grew rapidly within the first month and then developed slowly and tended to be steady [11,20,45]. The SCC had a highest autogenous shrinkage due to no expansive agent admixed, and the SF0 had a lowest autogenous shrinkage due to the free development of expansive deformation. With the increase of the *v*_f_ from 0.4% to 1.2%, the autogenous shrinkage of self-compacting SFRC compared with SCC at 180 d reduced by 22.2% to 3.2%. The change of autogenous shrinkage of self-compacting SFRC with a varying *v*_f_ is controlled by the complex effect of steel fibers. On the one hand, the rigid skeleton support of steel fibers reduces the autogenous shrinkage of self-compacting SFRC. On the other hand, the bridging effect of steel fibers confines the expansion of self-compacting SFRC. In this study, the autogenous shrinkage of self-compacting SFRC increased due to the weakness of aggregate skeleton with the increase of binder to coarse-aggregate ratio and sand ratio, while the expansion of self-compacting SFRC changed slightly with a constant dosage of expansive agent. Therefore, with the increase of the *v*_f_ from 0.4% to 1.2%, the expansion rate of self-compacting SFRC reduced 58.2% at the early 3 d, and the expansive time of SF12 was shorted as about half of SF4. Similar results were reported in literatures [28,29,30]. This led to the higher autogenous shrinkage before 90 d, and the autogenous shrinkage at 28 d of SF12 was about twice that of SF4. With the increase of curing age, however, the autogenous shrinkage of self-compacting SFRC decreased by the bridging effect of steel fibers strengthened with the increase of *v*_f_. In this study, the autogenous shrinkage of SF4 and SF8 still grew obviously after 90 d, but the autogenous shrinkage of SF12 tended to be steady, which led to the autogenous shrinkage of self-compacting SFRC decreasing with the increased *v*_f_ from 0.4% to 1.2% after 90 d.

### 3.3. Drying Shrinkage of Hardened Self-Compacting SFRC

**Figure 8:**
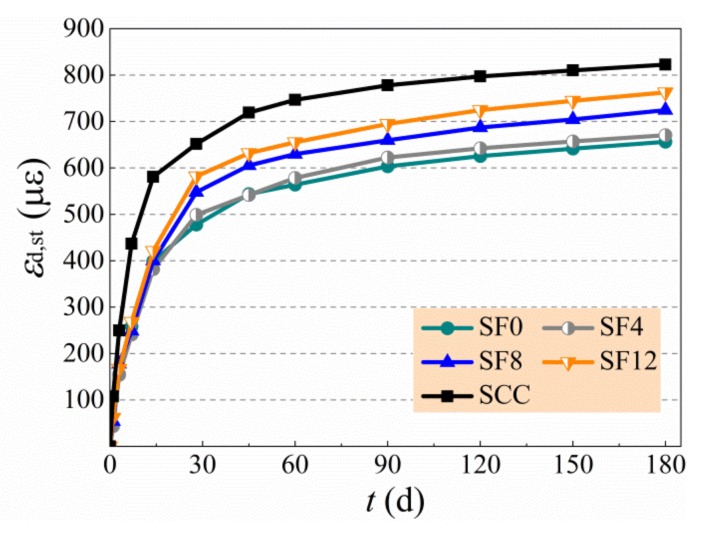
Drying shrinkage of tested specimens.

The test results of drying shrinkage of specimens are exhibited in Table 7 and Figure 8. After the commencement of drying, the shrinkage rate developed rapidly and more than 50% of shrinkage happened within the first month [8–10,12,45]. The drying shrinkage at 180 d of SF0 decreased 20.3% compared with SCC due to the compensation effect of expansive agent. With the increase of the *v*_f_ from 0.4% to 1.2%, the drying shrinkage at 180 d of self-compacting SFRC compared with SCC reduced by 18.5% to 7.3% due to the increase of inhibition effect of steel fibers. Meanwhile, the drying shrinkage of SF12 with the comparison of SF0 was increased by 16.3%. The change of drying shrinkage of self-compacting SFRC with a varying *v*_f_ was also influenced by the complex effect of binder materials and steel fibers. The increased binder to coarse-aggregate ratio and sand ratio had negative effects on the reduction of drying shrinkage of self-compacting SFRC due to the weakness of aggregate skeleton and the inevitable loss of free water from macro and micro pores of concrete. Oppositely, the steel fibers provided a rigid skeleton support to reduce the drying shrinkage of self-compacting SFRC. Generally, with the aid of expansive agent, the self-compacting SFRC had a reduced drying shrinkage than SCC, and an increased drying shrinkage with the volume fraction of steel fiber.

### 3.4. Compressive Strength

**Figure 9:**
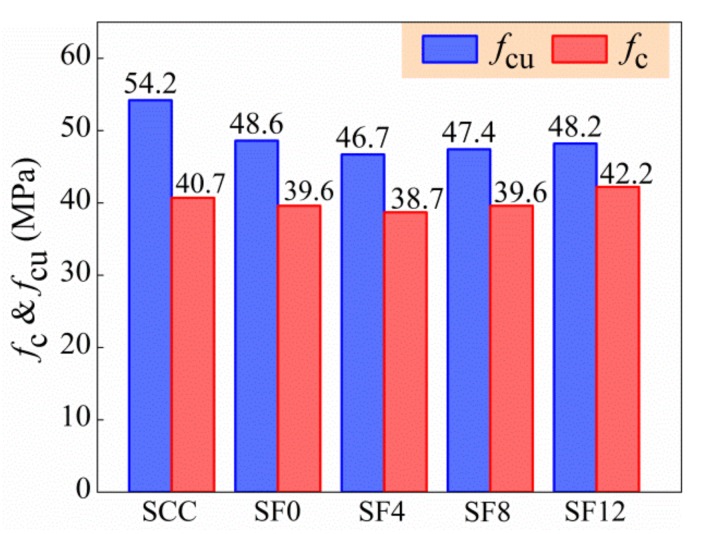
Compressive strength of tested specimens.

The test results of cubic compressive strength (*f*_cu_) and axial compressive strength (*f*_c_) of specimens are shown in Figure 9. All reached the target cubic compressive strength of 46.6 MPa. Due to the unconfined transversal expansion of specimens with the addition of expansive agent, the *f*_cu_ and *f*_c_ of SF0 compared with SCC decreased by 10.5% and 2.7%. This is similar as it was reported the expansive agent has more influence on cubic compressive strength than axial compressive strength of SCC [26,27]. With the increase of the *v*_f_ from 0.4% to 1.2%, the *f*_cu_ and *f*_c_ of self-compacting SFRC increased by 3.2% and 9.1%. This attributes to the inhibition effect of steel fiber on the unconfined transversal expansion of specimens; the cubic specimens were less inhibited than the cylinder specimens due to the different height of specimens. Differing from that the compressive strength of self-compacting SFRC reduced with the increase of the *v*_f_ reported [10,12,13,19], the compressive strength of self-compacting SFRC in this study trends to be increased with the incorporation of expansive agent and steel fibers.

### 3.5. Splitting Tensile Strength

The test results of splitting tensile strength (*f*_t_) of specimens are shown in Table 8. The splitting tensile strength of SF0 compared with SCC had a slight reduction of 3.5%, while the splitting tensile strength of self-compacting SFRC was improved obviously [17,19]. With the increase of the *v*_f_ from 0.4% to 1.2%, the *f*_t_ increased by 59.8%. This may be attributed to the self-prestress effect. The cementitious matrix expansion confined by steel fibers leads a reciprocal interfacial stress between fibers and matrix, which induces an internal uniform compressive prestress of matrix, and improves the chemical bond between steel fibers and cementitious matrix [30,46].

By using Equation (3) in China code JGT 472 [42], the tested values (*f*_t_) and calculated values (*f*_t,c_) are compared in Table 8. The mean value of *f*_t_/*f*_t,c_ was 0.948, with a dispersion coefficient of 0.071. This indicates that the Equation (3) can be used to predict the splitting tensile strength of self-compacting SFRC with expansive agent.

(3) $$\;f_{\mathrm{ft}}\;=\;f_{t}\left(1+0.76\lambda_{f}\right)\;$$

where, *f*_t_ is splitting tensile strength of SCC; *λ*_f_ is the fiber factor, i.e., the product of *v*_f_ and *l*_f_/*d*_f_.

### 3.6. Modulus of Elasticity

Test results of the modulus of elasticity (*E*_c_) of specimens are presented in Table 9. The value of SF0 was 10.3% lower than that of SCC. Due to the inhabitation of steel fibers to transverse deformation of self-compacting SFRC under axial compression, the modulus of elasticity increased with the *v*_f_. In this study, with the increase of the *v*_f_ from 0.4% to 1.2%, the *E*_c_ increased by 17.0%. With Equation (4) used for conventional concrete [47], the predicted values *E*_c,c_ of the modulus of elasticity of self-compacting SFRC are presented in Table 9. The mean ratio of *E*_c_/*E*_c,c_ is 1.125, with a dispersion coefficient of 0.056. A conservative predicted modulus of elasticity of self-compacting SFRC with expansive agent is given out by Equation (4).

(4) $$E=\;10^{5}/\left(2.2+34.7/f_{\mathrm{cu}}\right)\;$$

## 4. Conclusions

With the premise of ensured workability of a fresh mixture, this paper studied the effect of calcium-sulfoaluminate expansive agent on shrinkage and mechanical properties of hardened self-compacting SFRC with a varying volume fraction of steel fiber. Based on the test results, conclusions can be drawn as follows:

(1) The mix proportion of self-compacting SFRC with expansive agent can be designed by the direct absolute volume method, of which the steel fibers are considered as the distributed coarse aggregates. The rational workability of high filling and passing ability without segregation and bleeding of fresh self-compacting SFRC can be ensured by increasing the binder to coarse-aggregate ratio and the sand ratio in the mix proportions. With the increment of 0.4% volume fraction of steel fiber, the sand ratio increased by 0.01 and the binder to coarse-aggregate ratio increased by 0.10. The optimal content of calcium-sulfoaluminate expansive agent was 10% mass of binders.
(2) The autogenous shrinkage of self-compacting SFRC increased before 90 d and decreased after 90 d with the increase of volume fraction of steel fiber. With the incorporation of an expansive agent of 10% mass of binders and the steel fiber changed with the volume fraction from 0.4% to 1.2%, the autogenous shrinkage of self-compacting SFRC reduced by 22.2% to 3.2% at curing age of 180 d.
(3) The drying shrinkage of self-compacting SFRC reduced by 18.5% to 7.3% with the incorporation of expansive agent of 10% mass of binders and the steel fiber changed with the volume fraction from 0.4% to 1.2%. The expansion of cementitious matrix was reduced with the increase of volume fraction of steel fiber. This leads an increased drying shrinkage of self-compacting SFRC with the increase of volume fraction of steel fiber.
(4) The mechanical properties of self-compacting SFRC were improved with the incorporation of expansive agent and steel fiber. With the increase of a volume fraction of steel fiber from 0.4% to 1.2%, the cubic compressive strength, axial compressive strength and modulus of elasticity of self-compacting SFRC increased by 3.2%, 9.1% and 17.0%, respectively. At the same time, the splitting tensile strength of self-compacting SFRC was increased significantly by 59.8%.

## Figures and Tables

**Table 1:** Chemical compositions of binder materials.

Binder Materials	Chemical Compositions (%)								
	SiO_2_	Fe_2_O_3_	Al_2_O_3_	CaO	MgO	SO_3_	Na_2_O	K_2_O	LOI
Cement	20.81	3.28	5.99	60.12	2.13	2.23	0.11	0.55	3.52
Fly ash	55.92	5.91	17.31	6.59	3.82	1.93	0.48	1.96	2.63
Limestone powder	0.89	0.28	0.51	47.56	4.45	0.06	0.67	0.27	40.71
Expansive agent	3.48	3.12	11.27	42.78	0.48	27.38	0.62	0.47	4.51

**Table 2:** Physical and mechanical properties of ordinary silicate cement.

Density (kg/m^3^)	Fineness (m^2^/kg)	Setting Time (min)		Flexural Strength (MPa)		Compressive Strength (MPa)	
		Initial	Final	3d	28d	3d	28d
3093	360	170	215	5.4	8.3	27.5	55.6

**Table 3:** Physical and mechanical performances of fly ash and limestone powder.

Mineral Admixture	Density (kg/m^3^)	Fineness (m^2^/kg)	Water Demands Ratio (%)	Water Content (%)	Active Index (28d, %)
Fly ash	2342	406	84	0.1	73.3
Limestone powder	2779	428	−	0.2	61.2

**Table 4:** Mix proportion of SCC and self-compacting SFRC.

Mixture	SCC	SF0	SF4	SF8	SF12
*w/b*	0.31	0.31	0.31	0.31	0.31
Cement (kg/m^3^)	418	358	380	401	423
Fly ash (kg/m^3^)	60	60	63	67	70
Limestone powder (kg/m^3^)	119	119	127	134	141
Expansive agent (kg/m^3^)	0	60	63	67	70
Manufactured sand (kg/m^3^)	786	786	785	783	780
Crushed limestone (kg/m^3^)	852	852	777	703	631
Steel fiber (kg/m^3^)	0	0	31	63	94
Water (kg/m^3^)	185	185	196	207	218
Superplasticizer (kg/m^3^)	7.16	7.16	7.59	7.35	7.75
Binder materials (kg/m^3^)	597	597	633	668	704
Sand ratio (%)	48	48	49	51	52
Binder to coarse-aggregate ratio	0.71	0.71	0.78	0.87	0.97

**Table 5:** Workability of self-compacting SFRC.

Test Method	Index	SCC	SF0	SF4	SF8	SF12	Target Values
Slump-flow	*D* (mm)	710	720	710	705	710	>600
	*T*_50_ (s)	3.8	4.2	5.5	5.8	6.4	<8
J-ring	*D*_J_ (mm)	700	700	695	675	680	−
	*D*-*D*_J_ (mm)	10	20	25	30	30	<50

**Table 6:** Autogenous shrinkage of SCC and self-compacting SFRC.

Mixture	*ε*_as,t_ (με)										
	1 d	3 d	7 d	14 d	28 d	45 d	60 d	90 d	120 d	150 d	180 d
SCC	−4	43	103	141	185	212	228	247	249	250	252
SF0	−66	−84	−43	31.3	46	61	75	84	90	92	95
SF4	−66	−77	−34	22.6	61	100	127	174	205	229	244
SF8	−55	−64	−6	65.6	100	138	159	183	199	209	219
SF12	−29	−35	25	76	121	147	164	178	188	191	196

**Table 7:** Drying shrinkage of SCC and self-compacting SFRC.

Mixture	*ε*_ds,t_ (με)										
	1 d	3 d	7 d	14 d	28 d	45 d	60 d	90 d	120 d	150 d	180 d
SCC	108	250	437	581	652	719	747	778	792	810	823
SF0	44	155	259	399	478	544	564	603	626	642	656
SF4	44	154	241	382	499	542	578	622	642	657	671
SF8	54	178	249	400	548	605	630	660	687	704	725
SF12	62	167	268	421	582	632	655	695	725	745	763

**Table 8:** Tested and calculated values of the splitting tensile strength of specimens.

Mixture	SCC	SF0	SF4	SF8	SF12
*f*_t_ (MPa)	3.26	3.15	3.28	4.16	5.24
*f*_t,c_ (MPa)	−	3.26	3.85	4.45	5.04
*f*_t_/*f*_t,c_	−	0.97	0.85	0.94	1.04

**Table 9:** Tested and calculated values of the modulus of elasticity of specimens.

Mixture	SCC	SF0	SF4	SF8	SF12
*E*_c_ (GPa)	40.7	36.5	35.3	39.6	41.3
*E*_c,c_ (GPa)	35.2	34.3	34.0	34.1	34.3
*E*_c_/*E*_c,c_	1.16	1.06	1.04	1.16	1.20
